# Effects of attentional focus on the regulation of torque complexity

**DOI:** 10.1371/journal.pone.0325302

**Published:** 2025-06-25

**Authors:** Philipp Bauer, João S. Gomes, João H. Oliveira, Paulo Santos, Pedro Pezarat-Correia, João R. Vaz

**Affiliations:** 1 Egas Moniz Center for Interdisciplinary Research (CiiEM), Egas Moniz School of Health & Science, Caparica, Almada, Portugal; 2 Neuromuscular Research Laboratory, Faculty of Human Kinetics, University of Lisbon, Cruz-Quebrada, Lisbon, Portugal; 3 CIPER, Faculty of Human Kinetics, University of Lisbon, Cruz-Quebrada, Lisbon, Portugal; Shahid Chamran University of Ahvaz, IRAN, ISLAMIC REPUBLIC OF

## Abstract

Recent scientific evidence suggests that an external focus of attention (vs. an internal focus of attention) promotes a greater number of motor solutions, rendering the system more adaptable and complex. Therefore, the present study investigated the effect of the attentional focus (external vs. internal) on torque complexity and variability as well as its associated intermuscular coordination processes. Fourteen participants performed maximal and submaximal isometric knee extension tasks in three conditions (control, external focus, internal focus) to assess immediate effects of the focus of attention in a within-subjects comparison. Peak torque was extracted from maximal trials. Regarding submaximal trials, torque complexity and the magnitude of variability were assessed through Sample Entropy (SampEn), i.e., a measure of regularity in the temporal structure of torque output and coefficient of variation (CV), respectively. The intermuscular coordination was assessed through co-contraction index. An external focus led to an increase in SampEn (i.e., decreased regularity) when compared to an internal focus (p = 0.032) and a control condition (p = 0.036). Conversely, no differences were found for CV and peak torque. The external focus promoted a decrease in muscular activity of vastus medialis in comparison with the internal focus (p = 0.040) and an increase in muscular activity when compared to the control condition (p < 0.001). Furthermore, an external focus led to decreased muscular activity of semitendinosus as well as a decrease in the co-contraction indices involving semitendinosus in comparison with the internal focus and the control condition (all p < 0.05). The present findings suggest that an external focus leads to an enhanced flexibility of motor control. Moreover, the general decrease in co-contraction and in muscular activity without affecting maximal force parameters that we observed with an external focus suggest a higher efficiency of the motor system caused by intermuscular coordination processes.

## Introduction

In movement sciences, variability is characterized as the normal fluctuations across repetitions of a motor task [1]. Historically, there is a disagreement of theorists whether those variations are seen as noise (i.e., random error) [2] or as systematic (i.e., information-rich) [2,3]. Traditionally, these fluctuations are studied according to their magnitude through linear measures, namely the Coefficient of Variation (CV). However, these measures only provide a description of the amount of variability around a central point, assuming that fluctuations between repetitions of a task are random and independent of past and future repetitions. Previous studies have shown these fluctuations are distinguishable from noise [4–6] and possess a deterministic origin [5–8]. Thus, these fluctuations in the time series are neither random nor independent. Rather, the temporal structures of the physiological time-series contain hidden information, which differs from linear, magnitude-based measures of variability [9]. Accordingly, variability can be analyzed in two ways: linear and non-linear. To illustrate the difference between both variability analysis, Stergiou and colleagues [10] use the motor learning paradigm. According to the authors, with skill acquisition, there is a decrease in linear variability and an increase in non-linear variability towards an optimal state. This could be explained by the development of a richer behavioral repertoire, which increases the number of motor solutions available to individuals to perform a task. The difference of those two types of variability can be best illustrated in expert athletes as they show a low error rate in movement execution (i.e., low linear variability), but a large behavioral repertoire in movement choice (i.e., high non-linear variability).

Interestingly, Stergiou and colleagues [10] have proposed a theoretical model of optimal movement variability in which they suggest that a richer behavioral state is characterized by chaotic, non-random, yet structured fluctuations, rendering the system more complex. Specifically, a complex system is not purely regular, rigid and predictable; but neither is it random and unpredictable. Accordingly, the individual becomes more able to flexibly adjust the physiological outputs to intrinsic, environmental and task-related constraints [11]. A system’s complexity is affected by several factors, such as aging [12], disease [13], injury [14], fatigue [15], task modalities [16], circadian rhythms [17], genotype and surrounding circumstances [18]. Most of these factors are not alterable in real life scenarios of motor learning, while the surrounding circumstances can be easily adapted to promote changes in a system’s adaptability and, consequently, complexity [19].

Surrounding circumstances are controllable variables, for example, feedback instructions. Specifically, extrinsic feedback has been shown to increase retention in motor learning [20,21]. Extrinsic feedback can be provided as knowledge of results or knowledge of performance. Knowledge of results is characterized as information referring to the outcome of executing a skill or to the goal of the performance (i.e., external focus of attention), while knowledge of performance is defined as information characterizing the movement itself, which leads to the performance outcome (i.e., internal focus of attention) [22]. Van Vliet and Wulf [23] suggested that focusing externally enhances motor learning more than focusing internally. More recent findings from Gokeler and colleagues [24] support this assumption as focusing externally generates a flexible motor system, which easily adapts to changing environments and is less prone to injuries, enhancing motor skill acquisition. Furthermore, Kakvandi and colleagues [25] found that focusing externally not only enhances skill acquisition and motor learning in a single-task, but also show its transfer to a dual-task scenario, suggesting an increased movement automatization and efficiency.

The benefits of an external focus (EF) are thought to be explained by the behavior of muscular activity and neurophysiological mechanisms. In particular, the literature suggests that focusing externally typically leads to a more efficient muscular activity than focusing internally [26]. This phenomena has been shown in biceps curl exercises [27,28], sit up exercises [29] and knee extension exercises [30]. The findings of Marchant and Greig [30] in a maximal voluntary contraction task of knee extension state that there is no difference in performance of torque production, despite muscular activity being different between focus of attention conditions. In the EF condition individuals were able to produce the same torque output as in the internal focus (IF) condition, but with less muscular activity, which characterizes an increased efficiency. According to the constrained action hypothesis, an IF leads individual’s attention to their muscle/movement, which could actually constrain or perturbate automatic control processes, whereas an EF, by focusing on the movement goal, allows the motor system to self-organize in a more natural way [31,32]. Interestingly, studies using electroencephalography have shown that adopting an IF may result in real-time conscious motor processing, which leads to perturbation in the motor system [33,34]. This perturbation is evident in intermuscular coordination, as an IF is claimed to not only provoke unnecessary contractions of the agonist [35,36], but also of other surrounding muscles (i.e., agonist co-contraction) [28,36–38]. Importantly, compared to an EF, the IF is also associated with an increase in the antagonists muscular activity, leading to an increased antagonist co-contraction [39,40].

Furthermore, regarding maximal performance, despite Marchant and Greig [30] did not find differences in the maximal torque production with different focus of attention conditions, there is a solid body of literature indicating changes in maximal force production. Those studies indicate that an EF leads to increased maximal force production in maximum voluntary isometric contraction (MVIC) tasks on elbow torque [27], multi joint torque [41] and knee extension torque [42]. This advantage of an EF is thought to be based on the fact that an EF allows to find the optimal solution to execute the task, as an EF is known to “free” degrees of freedom and offer variability in terms of task execution [39,43]. Variability within movement execution emerges from a large behavioral repertoire in movement choice, which is inherent in a complex system [10].

This motivated us to investigate whether an EF strategy leads not only to benefits in the maximal performance of torque production [27,41,42], but more importantly in the regulation of torque production, increasing the capacity of the neuromuscular system to adapt force output to the task demands [10]. In addition, the present study aimed to explore the intermuscular coordination mechanisms that could explain the potential differences between focus conditions. We hypothesize that an EF, compared to a control condition and an IF, will lead to i) a less constrained torque regulation (i.e., increased entropy, a measure of non-linear variability), suggesting an increased torque complexity; ii) a decrease in CV and iii) an increased capacity of maximal force production. Regarding the underlying neurophysiological mechanisms, we hypothesize that an EF (vs. a control condition and an IF) will lead to iv) a lower electromyography (EMG) activation across agonists and antagonists and v) a decreased antagonist co-contraction index.

## Materials and methods

### Subjects

A priori sample size estimation determined that fifteen participants would provide 90% power to detect an effect size of η_p_^2^ = 0.14 using a one-way repeated measures ANOVA (CON, IF, EF) at a significance level of 0.05. Consequently, a total of 15 healthy male participants were recruited from the 1^st^ of February to the 31^st^ of March 2022 by word of mouth and tested in the present study. Due to technical issues, the collected data of one participant had to be excluded from further analysis. Therefore, the analyses were carried out based on the data of fourteen participants aged 20–29 years. Criteria of exclusion were severe cardiovascular or pulmonary disease, lower limb disabilities, neurological disorders and other orthopedic concerns, which might limit force production. Furthermore, the participants were instructed to avoid heavy exercise 48h and drugs, caffeine, tea or other stimulating substances 12h before the evaluation. Because this study implies conscious focus conditions, special attention was paid to not provide participants with information about the hypothesis or the expected outcome of the study to not influence the results. Prior to participation, all participants signed an informed consent approved by the ethics committee of the Faculty of Human Kinetics and in conformity with the Declaration of Helsinki. Risks in terms of health were minimal, since participants were supervised during the whole evaluation.

### Experimental design and protocol

Participants were asked to visit the laboratory once and perform a familiarization followed by three evaluation sessions. The rest times were defined as 5 min between sessions. The familiarization session consisted of a warm-up, three flexion MVICs, three extension MVICs and three submaximal sustained contractions. The first evaluation session was determined as control condition (CON), without focus instruction and consisted of three flexion MVICs, three extension MVICs and two submaximal sustained contractions. The focus conditions (IF and EF) were then randomly allocated to sessions two and three in which participants executed three extension MVICs and two submaximal sustained contractions. In all sessions, the rest between each trial was set to one minute.

**Fig 1:**
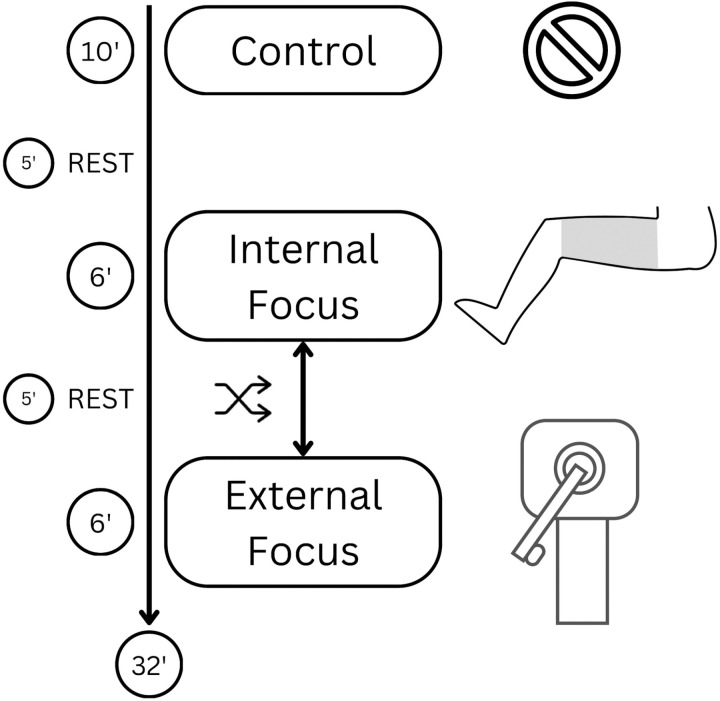
Infographic of the experimental design with the respective focus instructions (i.e., control- no focus instruction, internal focus- quadriceps, external focus- lever arm of the machine). The respective times are presented in minutes and include maximal as well as submaximal repetitions.

Common instructions were given in all sessions before each repetition. In the MVIC repetitions, participants were instructed to execute the movement the hardest and fastest possible without countermovement. During the execution, participants were strongly verbally encouraged to execute their maximum force. In the submaximal repetitions, it was explained that the researcher will place the participants’ leg in a certain position and that the goal is to maintain that position while viewing straight forward. In sessions two and three (focus condition sessions), the respective focus instructions were given verbally together with the common instructions before each repetition. In the internal focus condition, participants were instructed to focus on the contraction of their quadriceps, while in the external focus condition, they were instructed to focus on either pushing or resisting the lever arm of the machine (Fig 1). During the submaximal repetitions, the focus instruction was repeated every 10s.

### Data collection

**Fig 2:**
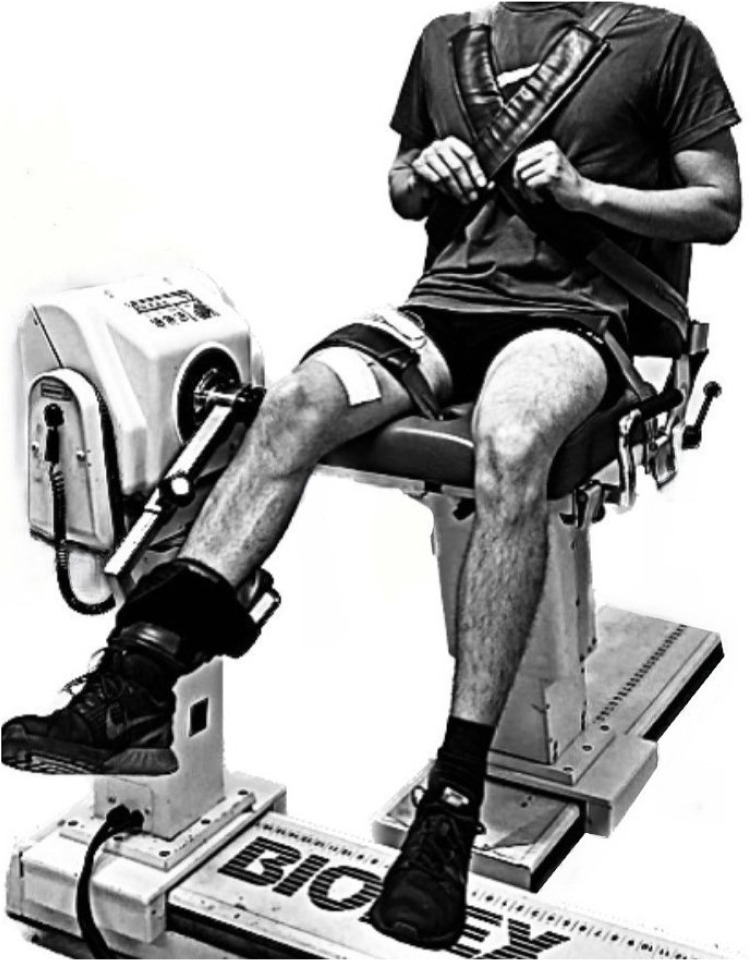
Experimental set up with the participant seated on the isokinetic dynamometer.

For the analyses the Biodex System 3 Pro Isokinetic Dynamometer (Biodex Medical Systems Inc., Shirley, NY) was used. Setting and calibration were performed according to the manufacturer’s information. Participants were seated so the lateral epicondyle of the femur was in line with the lever arm rotation axis and the knee as well as the hip angle were 90°. To maintain the position during contraction, participants were secured with straps around hip and shoulders. The dominant leg was strapped to the lever arm. All tasks were of isometric nature. Extension MVICs and submaximal contractions were realized at 70°, while Flexion MVICs were realized at 30° of knee flexion (Fig 2). In the MVICs, participants were asked to exert their maximum force for at least 4s and trials were repeated if a countermovement exceeded 7N.m. In the submaximal contractions, participants performed 40% of their extension MVIC of the control condition, independent on the round. All submaximal contractions were hold leg extension tasks realized without visual feedback to not influence the focus condition. Participants were asked to maintain their leg in position for 30s after stabilization at 70° of knee flexion and trials were immediately interrupted and repeated if the angle varied by more than 10°. No instructions for task correction were given to not influence the focus of attention. All signals were sampled with Biopax MP150 data acquisition and analysis system (Biopac Systems Inc., Goleta, CA) at 1000 Hz and interfaced with a personal computer. Data was collected with the software AcqKnowledge (Version 4.1.1., Biopac Systems Inc., Goleta, CA).

Muscle activation of rectus femoris (RF), vastus lateralis (VL), vastus medialis (VM), biceps femoris (BF) and semitendinosus (ST) was recorded with surface EMG (Delsys Trigno®, Delsys Inc., Natick, MA). To ensure maximal quality of the EMG signal, the skin was shaved and disinfected with alcohol before placement of the sensors. The electrodes were placed in line with the muscle fibers according to SENIAM (Surface EMG for Non-invasive Assessment of Muscles) [44]. The sensors were fixed with a special adhesive interface and additional tape to prevent alterations of position during movement. Signals were pre-amplified, band-pass filtered with 20–450 Hz and digitized at minimum 1259 Hz.

Participants’ reported thoughts during the submaximal task were assessed immediately after each evaluation round. The answers were written down literally without giving further information.

### Data analysis

Data analysis of all signals was realized with MATLAB® R2018a programming software (MathWorks, Natick, MA). Before analyzing the data for the variables of interest, the torque signal of the MVIC trials was low pass filtered (Butterworth 10 Hz, 4th order). The variable of interest in the MVIC trials was peak torque (PT), which equals the highest torque value of the trial. Selection criteria of the best MVIC trial, which was used for statistical analysis, were: the trial with the highest value of rate of torque development and that the PT of this trial needed to be at least 95% of PTmax of all three trials in the respective round.

In terms of the submaximal trials, the signal was kept in the raw format, no filtering was applied. Before the variability measures calculation, the torque signal was downsampled to 100 Hz after running a power spectral analysis that revealed an average maximal frequency across all participants of 18.31 Hz. Therefore, it was used the recommendation by Stergiou et al. [1] of a sampling frequency five times greater than the highest frequency in the time series of interest. This was conducted by analyzing the power spectrum density. Furthermore, the signals were cropped at the beginning and at the end to avoid the ascendant and descendant part of the contraction. The temporal structure of the torque output was analyzed using sample entropy (SampEn), which indicates the probability that short sequences of data points are repeated throughout the signal length [45]. Lower SampEn (towards 0) is the consequence of similar distances between repetitions of those sequences, which indicates a more regular torque output. A higher SampEn value (towards infinity) results from large differences of distances between repetitions of sequences, which designates a more irregular, variable torque output. For the determination of SampEn, in the present study the values were set as follows: pattern length (m) at 2, error tolerance (r) at 0.2 and data length (N) at 3000 data points (i.e., 100 Hz x 30s) [46]. SampEn measures showed the highest reliability when these input values were identical for the total of all trials [47]. The linear measure of variability was analyzed with the coefficient of variation (CV), which indicates the amount of variability within the torque signal. Furthermore, the mean torque was calculated as the average of torque output of each submaximal trial. Similarly, the mean angle was calculated as the average of the lever arms’ angular position during the contraction. SampEn, CV, mean torque and mean angle were calculated from the exact same cropped signal and for statistical analysis, the mean of the two submaximal trials of each focus condition was used.

Regarding EMG, raw data of the submaximal trials was band-pass filtered (20–490 Hz), rectified, smoothed (low-pass filter 12 Hz, 4th order Butterworth) and normalized to the highest value of the three MVIC trials of the control condition. Then, the EMG data was cropped at the beginning and at the end, so it only showed the time interval in which the participant matched with the target force. Variables of interest were the mean individual EMG amplitude and the co-contraction index (CCI) between the following pairs: VL/BF, VL/ST, VM/BF, VM/ST, RF/BF and RF/ST. CCI was determined based on Rudolph et al. [48]. The mean of the two submaximal trials of each focus condition was used for statistical analysis of the EMG parameters.

Moreover, based on participants’ given thoughts, two dominant thoughts analyses were carried out. The first analysis shows how well participants followed the focus instructions in the submaximal contractions of sessions two and three (i.e., focus condition sessions). Two coders classified the given thoughts independently either as proving that the focus instruction was followed or not. The given thoughts consist of a brief comment. The focus instruction was classified as ‘followed’, if at least one of the words in the comment was identical to the key word of the respective focus condition, i.e., the lever arm or machine for the EF and the muscle or quadriceps for the IF. The two coders agreed in 93% of the cases. For the cases in which they did not agree, a third coder was consulted to make the final decision. In the second analysis, just the comments regarding the submaximal contractions of session one (i.e., control session) were rated to understand what participants’ dominant thoughts without focus instruction were. Similar to the first analysis, three coders were involved. The first one determined clusters based on the key words of the participants’ comments and assigned one or more clusters to a comment. The key words of the participant’s comment are the most indicative nouns referenced within the comment. The criterion for the assignment of a cluster to a comment is the clusters’ key word being identical to a comments’ key word. The second coder assigned the comments to the previously determined clusters with 94% compliance to the first one. For the dissimilar assigned comments, the decision was made by the third coder.

### Statistical analysis

All statistical analyses were conducted with Jamovi (Version 1.6., Sydney, NSW) and significance was set to p < 0.05. To summarize the processed torque and EMG data, standard descriptive statistics, such as mean and standard deviation were used. The processed data was tested for normality with the Shapiro-Wilk test and for sphericity with Mauchly’s sphericity test. If normality was attributed, all torque and EMG variables were tested using a one-way repeated measures ANOVA (CON, IF, EF). If sphericity was not attributed, the Greenhouse-Geisser correction was used. In case of significant main differences, Tukeys’ tests were performed to assess post hoc comparisons. Effect sizes were calculated using partial eta squared (η_p_^2^) and for post hoc comparisons Cohen’s d. If normality was violated, Friedmans’ test with Kendall’s W as a measure of effect size and in case of significant main differences, Durbin-Conover pairwise comparisons were used. Given the hypotheses directional set, a one-tailed approach was considered.

## Results

### Maximum voluntary isometric contraction (MVIC)

For PT, based on the Shapiro-Wilk test and Mauchly’s sphericity test, normality (all p > 0.05) and sphericity (p = 0.900) were confirmed. No significant differences were found between focus conditions (F_(2,26)_ = 2.34, *p* = 0.116, η_p_^2^ = 0.153).

### Submaximal contraction

**Fig 3:**
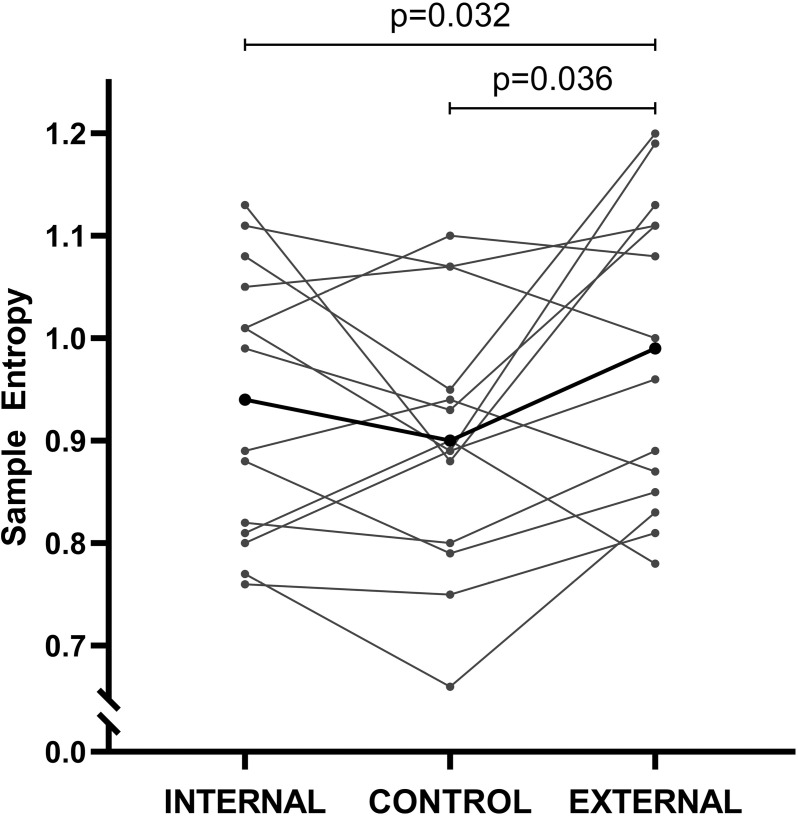
Torque sample entropy (AU) in the three conditions: Internal, control, external. Data is presented as single data points and means (i.e., dots in bolt). Comparisons were made using one-way repeated measures ANOVA (CON, IF, EF) with Tukey post hoc comparisons.

Assumption tests for mean torque revealed normality (all p > 0.05), but a violation of the assumption of sphericity (p = 0.001). Therefore, the Greenhouse-Geisser correction was used for mean torque. Normality (all p > 0.05) and sphericity (p = 0.268) were confirmed for mean angle. No significant differences were identified for mean torque (F_(1.20,15.64)_ = 2.05, *p* = 0.171, η_p_^2^ = 0.136) and mean angle (F_(2,26)_ = 1.13, *p* = 0.340, η_p_^2^ = 0.080). Regarding SampEn, the data conformed to the necessary statistical assumptions, ensuring the reliability of the analysis (all p > 0.05) and a significant main effect was found between focus conditions (F_(2,26)_ = 4.70, *p* = 0.018, η_p_^2^ = 0.266). Post hoc comparisons showed a higher SampEn with EF compared to CON (*p* = 0.036, d = 0.59) and with EF compared to IF (*p* = 0.032, d = 0.34), but no significant differences between CON and IF (*p* = 0.207, d = 0.26) (Fig 3). For CV, a normal distribution of the data in the control condition cannot be confirmed (p = 0.024). Therefore, Friedmans’ test was used, which showed no significant differences between focus conditions(χ^2^_(2)_ = 1.86, *p* = 0.395, W = 0.066) (Table 1).

**Table 1:** Coefficient of Variation (%), Peak Torque (N.m), Muscle Activation (%MVIC) and Co-Contraction Index of the analysed muscles in the three conditions: Control, external, internal.

	Control	External Focus	Internal Focus
Coefficient of Variation	5.04 ± 0.92	4.64 ± 0.83	4.88 ± 0.83
Peak Torque	304 ± 66.3	293 ± 61.5	287 ± 61.8
Muscle Activation			
Vastus Lateralis	26.6 ± 8.73†	28.4 ± 11.0	28.6 ± 9.31
Vastus Medialis	24.5 ± 7.39*†	27.9 ± 10.2#	28.2 ± 8.46
Rectus Femoris	19.6 ± 7.02	21.2 ± 9.37	20.8 ± 9.02
Biceps Femoris	2.59 ± 1.38*†	2.90 ± 1.65	3.42 ± 2.43
Semitendinosus	1.51 ± 1.20*†	1.38 ± 0.67#	1.78 ± 1.36
Co-Contraction Index			
Vastus Lateralis-Biceps Femoris	2.96 ± 1.71†	3.40 ± 2.15	4.21 ± 3.70
Vastus Lateralis-Semitendinosus	1.70 ± 1.59*†	1.48 ± 0.75#	2.03 ± 1.79
Vastus Medialis-Biceps Femoris	3.00 ± 1.75†	3.42 ± 2.20	4.21 ± 3.71
Vastus Medialis-Semitendinosus	1.72 ± 1.64*†	1.48 ± 0.75#	2.03 ± 1.83
Rectus Femoris-Biceps Femoris	3.07 ± 1.82	3.46 ± 2.22	3.86 ± 2.66
Rectus Femoris-Semitendinosus	1.73 ± 1.61*†	1.51 ± 0.77#	2.10 ± 1.84

Data is presented as mean values with standard deviation. Significant differences between the control and the external focus condition (*), the control and the internal focus condition (†) as well as the external and the internal focus condition (#) are indicated. Comparisons were made using one-way repeated measures ANOVA (CON, IF, EF) with Tukeys’ post hoc comparisons or the non-parametric alternative. p < 0.05.

Regarding muscular activation, data of VL showed normality (all p > 0.05), but no sphericity (p = 0.035), which justifies the use of the Greenhouse-Geisser correction. Data of VM, BF and ST showed violations (p < 0.05) of the normality assumptions, which suggests the use of Friedmans’ test. Muscular activation in VL (F_(1.40,18.21)_ = 4.15, *p* = 0.045, η_p_^2^ = 0.242), VM (χ^2^_(2)_ = 16.0, *p* < 0.001, W = 0.571), BF (χ^2^_(2)_ = 7.37, *p* = 0.025, W = 0.263) and ST (χ^2^_(2)_ = 15.9, *p* < 0.001, W = 0.568) showed significant differences between focus conditions. Post hoc comparisons on VL revealed significantly more muscular activity with IF compared to CON (*p* = 0.001, d = 0.21), but no significant differences between CON and EF (*p* = 0.076, d = 0.17) as well as EF and IF (*p* = 0.483, d = 0.02). In terms of VM, post hoc comparisons demonstrated significantly more muscular activity with EF compared to CON (*p* < 0.001, d = 0.37), with IF compared to EF (*p* = 0.040, d = 0.03) and with IF compared to CON (*p* < 0.001, d = 0.45). Post hoc comparisons on BF identified significantly more muscular activity with EF (*p* = 0.039, d = 0.20) and IF (*p* = 0.003, d = 0.40) compared to CON, but no significant differences between EF and IF (*p* = 0.123, d = 0.24). Regarding ST, post hoc comparisons revealed significantly more muscular activity with CON compared to EF (*p* = 0.002, d = 0.13), with IF compared to CON (*p* < 0.001, d = 0.20) and with IF compared EF (*p* = 0.010, d = 0.36). For RF activity, statistical test assumptions were confirmed (all p > 0.05) and no significant differences between focus conditions were found (F_(2,26)_ = 2.43, *p* = 0.108, η_p_^2^ = 0.158).

As the Shapiro-Wilk test revealed violations of normality (p < 0.05) for the following CCIs, Friedmans’ test was used for further analyses. The CCIs of VL and BF (χ^2^_(2)_ = 6.14, *p* = 0.046, W = 0.219), VL and ST (χ^2^_(2)_ = 14.7, *p* < 0.001, W = 0.525), VM and BF (χ^2^_(2)_ = 6.14, *p* = 0.046, W = 0.219), VM and ST (χ^2^_(2)_ = 15.5, *p* < 0.001, W = 0.554) as well as of RF and ST (χ^2^_(2)_ = 15.5, *p* < 0.001, W = 0.554) showed significant differences between focus conditions. Post hoc comparisons on the index of VL and BF identified significantly more co-contraction with IF compared to CON (*p* = 0.007, d = 0.42), but no significant differences between CON and EF (*p* = 0.055, d = 0.22) as well as EF and IF (*p* = 0.156, d = 0.26). In terms of the index of VL and ST, post hoc comparisons revealed significantly more co-contraction with CON compared to EF (*p* = 0.001, d = 0.17), with IF compared to CON (*p* < 0.001, d = 0.19) and with IF compared to EF (*p* = 0.038, d = 0.38). Post hoc comparisons regarding the index of VM and BF demonstrated significantly more co-contraction with IF compared to CON (*p* = 0.007, d = 0.40), but no significant differences between CON and EF (*p* = 0.055, d = 0.20) as well as EF and IF (*p* = 0.156, d = 0.25). In regard to the index of VM and ST, post hoc comparisons revealed significantly more co-contraction with CON compared to EF (*p* = 0.001, d = 0.18), with IF compared to CON (*p* < 0.001, d = 0.17) and with IF compared to EF (*p* = 0.019, d = 0.38). Similarly, post hoc comparisons on the index of RF and ST identified significantly more co-contraction with CON compared to EF (*p* = 0.001, d = 0.17), with IF compared to CON (*p* < 0.001, d = 0.20) and with IF compared to EF (*p* = 0.019, d = 0.40). The CCI of RF and BF showed a normal data distribution (all p > 0.05), but no sphericity of the data (p = 0.005). The ANOVA with Greenhouse-Geisser correction showed no significant differences between focus conditions (F_(1.27,16.45)_ = 2.33, *p* = 0.142, η_p_^2^ = 0.152). All mean values with standard deviation for the EMG results are shown in Table 1.

### Dominant thoughts analyses

The first analysis showed that participants’ compliance with the focus instructions was high as 88% of the comments were judged as evidencing that the focus instruction was followed and no participant was failing compliance in both focus conditions. In the second analysis, coders identified up to three thoughts (i.e., clusters) per participant with the most frequent one (39% of the answers) being the position of the leg or the position in general. Since the instruction in the control condition was to maintain the position, the cluster position cannot be seen as a bias. However, participants showed a tendency on focusing more frequently on the muscle (17%) instead on the lever arm (11%). Therefore, the control condition is assumed to not be entirely neutral in terms of focus of attention. The other given thoughts were similarly frequent (6–11%).

## Discussion

This study aimed to investigate the effect of the type of attentional focus on the regulation of torque complexity and the maximal force capacity. Additionally, it aimed to investigate the intermuscular coordination processes involved in the different conditions. We hypothesize that an EF, compared to a control condition and an IF, will lead to i) a less constrained torque regulation (i.e., increased sample entropy), suggesting an increased torque complexity; ii) a decrease in CV and iii) an increased capacity of maximal force production. Regarding the underlying neurophysiological mechanisms, we hypothesize that an EF (vs. a control condition and an IF) will lead to iv) a lower electromyography (EMG) activation across agonists and antagonists and v) a decreased antagonist co-contraction index.

First and foremost, we did not verify significant differences in the capacity of maximal force production (i.e., peak torque) between conditions. Similarly, Marchant and Greig [30] state that an EF, when compared to an IF, causes no significant differences in PT. Although we did not observe an effect of attentional focus on the capacity of maximal force production, we did find a significant effect on torque regulation.

Comparing the external with the internal focus condition, the present results confirm the hypothesis that adopting an EF leads to significantly increased torque SampEn (i.e., decreased torque regularity) when compared to an IF. These findings are in line with the constrained action hypothesis, which states that with an IF automatic control processes are disrupted, because of active disturbance in the natural organization of the motor system [31,32]. By instructing internally to focus on one muscle, the task execution is restricted to one possible solution. On the contrary, focusing externally allows the system to self-organize and introduces flexibility to find the best solution to execute the task. By adopting an EF, participants show flexibility and less regularity in the torque-time curves until a certain extent, leading to a somewhat more complex torque output. The extent of the advantage in complexity of an EF compared to an IF remains unclear, because as yet there are no reference values of torque complexity, inferred from sample entropy. To create a reference for complexity, the control condition was implemented in the study design. Based on the constrained action hypothesis, in contrast to the internal focus condition, neither the control nor the external focus condition are considered constrained in terms of solutions to execute the task, while an EF especially promotes flexibility of solutions as the system has the ability to self-organize and find the best motor solution for the task goal (i.e., automatic control processes) [23]. Therefore, entropy values of the control condition were thought to be close to, but significantly lower than entropy values of the external focus condition and significantly increased compared to the internal focus condition. However, the present results did not confirm this assumption, as SampEn in the control condition was significantly lower than in the external focus condition, but showed no significant differences from the internal focus condition. The reason for these results is most likely the bias of participants’ dominant thoughts revealed in the second dominant thoughts analysis. This analysis shows that in the control condition participants tend to focus on the quadriceps more than on other aspects. Therefore, the control condition cannot be seen as reference for complexity. Conversely, biases caused by different task settings in the respective conditions can be excluded as there were no significant differences in mean torque and mean angle.

Stergiou and colleagues [10] suggest that the regularity of a time-series (i.e., non-linear variability) provides distinct information from the magnitude-based measures of variability (i.e., linear variability). Regarding linear torque variability, it was hypothesized that an EF would lead to a decrease in CV compared to a control condition and an IF. This hypothesis cannot be confirmed in the present study, as there were no significant differences in CV between conditions. However, non-linear measures reflect focus of attention induced changes, since we found significant differences in SampEn (i.e., the temporal structures of the physiological time-series) between an external and internal focus condition. That suggests that the temporal structure of the torque time-curve possesses a deterministic order, which changes based on certain constraints and affects the system’s flexibility. Therefore, the present results support the theory of Stergiou and colleagues [10] that novel non-linear measures offer further and more meaningful information compared to traditional linear measures. Furthermore, the authors explain that a system with the optimal amount of non-linear variability is characterized by a rich behavioral repertoire of movement patterns.

In studies of dart-throwing, Lohse and colleagues [35,39] refer to this rich behavioral repertoire of movement patterns as functional variability. The authors state that an EF increases functional variability of the movement pattern, but reduces outcome variability on the dartboard. The explanation for this pattern is most likely that an EF “frees” degrees of freedom with the opportunity of exploring multiple movement options and carry out compensatory corrections in the kinetic chain [43,49]. Based on the present findings, adopting an EF increases torque complexity (i.e., decreased torque regularity) possibly through an increased number of degrees of freedom within the system. This explains why adopting an EF instead of an IF during the execution of sport skills helps to adapt to different environmental circumstances, to prevent injuries and to increase outcome accuracy [19,35,39]. Therefore, the present results are important in the fields of coaching, physical preparation and sports medicine. The literature states that a loss of torque complexity, is associated with a compromised motor control [9,50–53]. Moreover, a loss of torque complexity is related to aging [12,54], disease [13,55,56] and injuries [14]. Therefore, those populations tend to present a deteriorated motor control. Importantly, a substantial loss of motor control in the lower limbs is a great risk factor for falls in elderly, especially during acute disease [57]. Therefore, it is important to prevent this loss of motor control because falls can lead to the loss of independence in daily life. Based on the present findings, adopting an EF instead of an IF increases torque complexity, which potentially prevents the loss of motor control. Therefore, the present findings are important for prevention and rehabilitation programs with elderly or with individuals presenting the above-mentioned clinical conditions.

In addition to understanding the effects of an EF on motor control, it is important to recognize the mechanisms behind this benefit. It was hypothesized that an EF would lead to a lower EMG activation across agonists and antagonists, relative to a control condition and an IF. Regarding the muscular activity during the focus of attention conditions, this hypothesis was partially verified. For the agonists’ activity during the submaximal trials, a decreased activity was observed with EF compared to IF for VM; and a similar trend, although not significant, for VL. For the muscular activity of RF, no significant differences and a trend different from the other agonists were observed. That is probably due to the fact that it is a multi-joint muscle crossing hip and knee, while the vastus muscles only act on the knee joint. Since the evaluation task was mono-articular on the knee joint, involuntary contractions of RF around the hip joint could have resulted in irrelevant muscular activity, which may explain the different trend of RF. In the antagonists, an EF (vs. IF) resulted in significantly less muscular activity in ST and a tendency in the same direction was found for BF. Muscular activity in the control condition was hypothesized to be higher than in the external focus condition, but lower than in the internal focus condition. Surprisingly, this hypothesis was only verified for ST, while the rest of the analyzed muscles showed the least muscular activity in the control condition with all muscles, except of RF, showing significant differences to the internal focus condition. That goes against the findings of the second dominant thoughts analysis, which revealed that participants in the control condition tend towards an IF. However, it is known that the focus of attention in general affects muscular activity [28]. It could be speculated that in the external as well as the internal focus condition the verbal instruction itself could have increased awareness, activity of the sympathetic nervous system and muscular activity when compared to the control condition.

A greater decrease in muscular activity of the antagonists compared to the agonists, accompanying an EF (vs. an IF), indicates decreased antagonist co-contraction. In fact, it was hypothesized that an EF would lead to decreased antagonist co-contraction index. Regarding the focus of attention conditions, this hypothesis can be verified for the CCIs with ST, as significantly less co-contraction of all three evaluated quadriceps muscles with ST was found when adopting an EF compared to an IF. A tendency with the same pattern was shown for the quadriceps muscles with BF. Antagonist co-contraction is crucial for joint stability and stiffness in voluntary isometric contractions, such as isometric prehension [58]. We can observe this phenomenon in the present study, as an increased antagonist co-contraction leads to an increased regularity of the torque output (i.e., decreased SampEn), suggesting a constraint in terms of the joint’s degrees of freedom when focusing internally compared to externally. The fact that the CCIs of the quadriceps muscles showed significances with ST, but not with BF, can be explained by the following mechanisms: ST (vs. BF) shows generally more muscular activity [59] and is a more superficial muscle, which means muscular activity is easier to detect with surface EMG. The CCI in the control condition was hypothesized to be higher than in the external focus condition, but lower than in the internal focus condition. Interestingly, the CCIs involving ST follow this pattern, which is due to changes in muscular activity of ST between conditions. It could be speculated that biases, such as the manipulated control condition or increased sympathetic activity, are not influencing ST, but mainly the agonist muscles, as those are the prime actor leading to task performance. However, a limitation of the explained EMG results is that the significant differences for muscular activity and CCI are often small when analyzing the mean values and effect sizes of the post-hoc comparisons (i.e., d below 0.5), even though the effect sizes of the ANOVAs and the Friedman tests are generally large (i.e., η_p_^2^ and Kendall’s W above 0.14 and 0.5, respectively, [60]).

The present study shows that by adopting an external focus, compared to an internal focus, the behavior of muscular activity and neurophysiological mechanisms lead to an enhanced complexity in a system’s torque output. However, this gain of torque complexity does not influence performance in maximal force production tasks. In future studies, it would be interesting to assess the effect of the focus of attention on an isotonic exercise or on a whole strength training program and to extrapolate this effect to practical applications, such as sport skills or physical therapy. Furthermore, the literature states a distance effect of the focus of attention in sport skills and balance tasks. More distal external foci show a greater effectivity of performance and a greater automaticity of the movement than more proximal external foci [31,61–64]. In future studies, it would be of interest to assess if the distance effect of the focus of attention can be applied to torque complexity. If so, then a more distal external focus (e.g., feedback on a screen) would be expected to increase the magnitude of the present results.

## Supporting information

S1 File Full data set. This file contains the full data set for all variables assessed in the present study.
(PDF)
