# Supplementary material for: Effects of attentional focus on the regulation of torque complexity
Source: PLoS One. 2025 Jun 25;20(6):e0325302. doi: 10.1371/journal.pone.0325302 (PMC12192082; doi:10.1371/journal.pone.0325302)
Supplement: S1 File — This file contains the full data set for all variables assessed in the present study. (PDF) [file pone.0325302.s001.pdf]

Peak Torque

CON	EF	IF
410.89	406.12	363.36
321.37	331.47	304.41
242.11	294.79	276.18
214.85	209.26	248.23
331.09	326.4	334.65
363.57	350.66	340.48
337.17	288.18	296.94
200.44	167.28	149.07
349.87	289.75	367.58
233.79	242.8	209.93
243.99	250.94	234.91
304.59	291.06	265.43
385.47	349.33	330.47
320.25	307.1	299.2

Sample Entropy

CON	EF	IF
0.95	1.2	1.08
0.79	0.85	0.88
0.89	0.96	0.8
0.8	0.89	0.82
1.07	1	1.05
0.75	0.81	0.76
0.88	1.13	1.13
1.07	1.11	1.11
1.1	1.08	1.01
0.93	1.11	0.99
0.89	1.19	1.01
0.94	0.87	0.89
0.66	0.83	0.77
0.9	0.78	0.81

Coefficient of Variation

CON	EF	IF
6.73	6.43	6.35
6.71	5.72	5.64
4.28	4.68	4.67
4.28	3.89	3.66
4.65	4.69	4.67
4.89	3.86	4.4
4.87	5.21	5.92
5.54	5.47	6.03
4.17	3.89	3.77
4.83	4.36	4.77
6.32	4.94	6.33
4.63	4.24	4.05
4.77	3.61	3.89
3.96	4	4.1

Mean Torque

CON	EF	IF
163.72	163.81	164.59
127.23	127.61	127.88
96.8	96.93	97.34
83.43	83.41	83.54
131.05	132.95	131.9
138.66	144.8	144.98
134.19	133.81	134.19
78.53	80.4	79.04
139.12	139.18	138.39
91.97	92.93	92.78
96.44	97.09	97.74
123.53	122.32	121.7
152.19	152.88	153.21
128.48	127.48	127.82

Mean Angle

CON	EF	IF
66.46	67.06	66.92
68.12	67.11	72.24
68.65	70.44	67.55
68.27	68.59	66.33
67.41	67.64	65.63
69.45	65.68	63.96
68.75	70.1	68
67.85	69.79	71.19
65.97	65.74	65.98
69.13	68.93	68.7
71.36	72.04	71.34
65.95	68.6	68.23
67.76	70.17	69.66
71.89	74.65	68.35

Muscular Activity RF

CON	EF	IF
24.92	30.66	29.71
18.02	16.79	14.41
14.71	14.02	14.17
6.76	5.67	7.09
32.34	32.64	34.78
18.32	19.28	22.15
19.98	21.99	23.67
22.92	32.09	27.3
18.47	18.79	18.43
14.61	13.42	11.82
33.05	40.17	38.19
18.39	18.3	18.66
18.63	18.71	17.14
13.6	14.42	13.61

Muscular Activity VL

CON	EF	IF
37.45	45.84	41.18
20.18	20.3	24.87
23.08	22.06	23.66
29.96	32.16	31.1
32.39	35.42	36.95
25.01	25.66	28.2
22.84	17.49	22.68
18.03	19.71	17.99
23.87	27.7	23.55
20.01	23.73	21.62
48.62	54.53	50.92
25.54	24.97	27.4
30.53	32.46	32.4
15.13	15.25	17.76

Muscular Activity VM

CON	EF	IF
37.15	45.13	40.25
20.45	22.57	28.37
19.44	19.26	21.35
19.07	19.96	20.73
31.6	36.43	36.55
23.08	24.13	26.68
27.92	26.75	34.9
17.06	22.71	19.51
22.05	27.55	23.7
15.97	20.33	19.09
39.87	52.56	45.33
26.57	28.86	32.33
24.94	26.48	26.5
18.23	18.23	19.62

Muscular Activity BF

CON	EF	IF
2.24	2.62	2.45
1.8	1.74	3.88
1.21	1.14	1.24
4.15	4.53	4.25
0.23	0.27	0.27
5.32	5.4	5.83
4.24	3.28	3.69
3.5	5.03	4.08
1.46	1.66	1.71
3.15	5.39	10.26
2.5	3.13	3.02
1.36	1.49	1.65
2.52	2.38	3.03
2.53	2.53	2.5

Muscular Activity ST

CON	EF	IF
1.21	1.44	1.32
1.22	1.24	1.85
0.93	0.88	0.98
0.5	0.52	0.53
0.96	0.99	1.06
2.54	2.61	2.8
2.33	2.09	3.77
0.75	0.97	0.88
1.33	1.52	1.46
0.12	0.16	0.23
1.55	1.91	1.72
1.05	1.19	1.31
5.04	2.12	5.3
1.63	1.73	1.74

CCI VL-BF

CON	EF	IF
2.38	2.78	2.6
1.96	1.89	4.94
1.28	1.2	1.3
4.74	5.19	4.85
0.24	0.28	0.27
6.49	6.59	7.08
5.07	3.94	4.34
4.21	6.38	5.04
1.56	1.78	1.87
3.66	7.07	15.44
2.64	3.31	3.21
1.43	1.59	1.75
2.78	2.57	3.39
2.97	2.96	2.87

CCI VL-ST

CON	EF	IF
1.25	1.49	1.36
1.29	1.32	2
0.97	0.92	1.02
0.51	0.53	0.54
0.99	1.02	1.1
2.81	2.89	3.09
2.58	2.36	4.43
0.78	1.02	0.93
1.41	1.61	1.56
0.12	0.16	0.24
1.6	1.98	1.79
1.09	1.25	1.38
6.6	2.29	7.02
1.82	1.93	1.92

CCI VM-BF

CON	EF	IF
2.39	2.78	2.61
1.96	1.88	4.79
1.29	1.21	1.31
5.1	5.62	5.17
0.24	0.28	0.27
6.59	6.67	7.15
4.93	3.71	4.12
4.26	6.19	4.98
1.57	1.78	1.88
3.8	7.34	15.47
2.67	3.32	3.23
1.43	1.57	1.73
2.83	2.61	3.46
2.9	2.89	2.84

CCI VM-ST

CON	EF	IF
1.25	1.49	1.36
1.29	1.32	1.98
0.98	0.93	1.03
0.52	0.53	0.54
0.99	1.02	1.1
2.83	2.9	3.11
2.54	2.26	4.31
0.78	1.01	0.92
1.41	1.61	1.56
0.12	0.16	0.24
1.62	1.98	1.79
1.09	1.24	1.36
6.84	2.33	7.25
1.79	1.9	1.9

CCI RF-BF

CON	EF	IF
2.46	2.86	2.66
1.99	1.93	3.39
1.32	1.24	1.35
5.11	5.18	5.44
0.24	0.28	0.27
6.93	6.99	7.43
5.25	3.82	4.36
4.06	5.86	4.71
1.59	1.84	1.92
3.87	7.7	10.64
2.71	3.39	3.28
1.46	1.62	1.8
2.97	2.72	3.74
3.04	3	3

CCI RF-ST

CON	EF	IF
1.27	1.52	1.38
1.3	1.34	2.38
0.99	0.94	1.05
0.56	0.59	0.59
0.99	1.03	1.11
2.91	2.98	3.17
2.62	2.31	4.45
0.77	1	0.91
1.43	1.65	1.59
0.12	0.16	0.24
1.63	2	1.81
1.11	1.27	1.41
6.68	2.43	7.28
1.85	1.95	1.98
